# Nostalgia evocation through seasonality-conscious purchasing behavior revealed by online survey using vegetable names

**DOI:** 10.1038/s41598-022-09485-2

**Published:** 2022-04-02

**Authors:** Naomi Gotow, Yuko Nagai, Taro Taguchi, Yuko Kino, Hiroyuki Ogino, Tatsu Kobayakawa

**Affiliations:** 1 Human Informatics and Interaction Research Institute, National Institute of Advanced Industrial Science and Technology (AIST), Tsukuba Central 6, 1-1-1 Higashi, Tsukuba, Ibaraki 305-8566 Japan; 2 Kagome Co., Ltd., 17 Nishitomiyama, Nasushiobara-shi, Tochigi 329-2762 Japan

**Keywords:** Psychology, Human behaviour

## Abstract

Food can be a valuable mediator of nostalgia. Japanese food culture places a great emphasis on seasons, and thus there may be a relationship between nostalgic foods and seasonality. In this study, we hypothesized that participants who emphasized seasonality at the time of vegetable purchase (seasonality-oriented; hereafter, SO) would be more likely to feel vegetable-evoked nostalgia than those who did not (non-seasonality-oriented; hereafter, non-SO). To test this hypothesis, we administered an online survey to older Japanese adults regarding 103 representative vegetables considered to be seasonal foods. After participants selected vegetables that they had eaten, they then selected those that evoked nostalgia (hereafter, nostalgic vegetables). For each nostalgic vegetable, they evaluated the degree of nostalgia and state if a past event (autobiographical memory) was recalled. If an autobiographical memory was recalled for a certain nostalgic vegetable, nostalgia for that memory was evaluated. Comparing between SO and non-SO participants, SO participants had significantly higher numbers of nostalgic vegetables with associated autobiographical memories, as well as nostalgia for those vegetables and autobiographical memories. The results supported our hypothesis, suggesting that seasonality-conscious purchasing behavior evokes nostalgia.

## Introduction

### Food seasonality and its psychological effects

Seasonality refers to the tendency of a time series to persistently repeat a yearly pattern or cycle^1^. Food seasonality can be a deciding factor in consumer dietary behavior^2^. However, the characterization of seasonality is not simple, and the definition of the term “seasonal food” varies depending on who uses it and the context in which it is used^3,4^. A survey funded by the United Kingdom’s Department for Environment, Food and Rural Affairs (DEFRA)^5^ used two definitions of seasonal food. The first definition is based on the global connection between production and consumption: “Food that is outdoor grown or produced during the natural growing/production period for the country or region where it is produced. It need not necessarily be consumed locally to where it is grown.” For example, apples grown and harvested during growing season in New Zealand are eaten in Europe during the spring and summer seasons^3^. This concept has been referred to as the production-based definition^4^, production-oriented approach^6^, or global seasonality^3,4,6^. The second definition by DEFRA is based on the local connection between production and consumption: “Food that is produced and consumed in the same climatic zone without high energy use for climate modification or storage.” For example, in Europe, apples are grown and harvested during the summer and autumn seasons, and eaten in October^3^. This concept has been referred to as the production and consumption-based definition^4^, consumer-oriented approach^6^, or local seasonality^3,4,6^. This study dealt with local seasonality from the consumer’s perspective.

Seasonal variations in patterns of food consumption tend to be more noticeable in temperate climates where seasonal variation in climate (specifically temperature) is more pronounced^7^. Most of the Japanese archipelago belongs to the temperate zone, and there are four distinct seasons (spring, summer, autumn, and winter) with marked temperature differences^8^. Therefore, Japanese cuisine, or “*washoku*,” places a strong emphasis on the seasonality of ingredients, and vegetables in particular are a fundamental element of *washoku*^9^. This Japanese awareness of seasonal vegetables is reflected in product displays in grocery stores and in the purchasing behavior of consumers. For example, many supermarkets place fresh vegetables in locations encountered by consumers as soon as they enter the store^10^. Approximately 50 types of vegetables are sold in supermarkets in Japan at any given time, although this number often exceeds 100^11^. According to the All Japan Supermarket Association^12^, there are three reasons for placing the vegetable section near the store entrance. The first is because vegetables are purchased more frequently than other foods. It is rare to find a menu that uses no vegetables, even meat or fish dishes prepared at home. The second is so that consumers can formulate a menu based on the vegetables they have selected. Many consumers come to the store without having first decided on a menu. The third is to motivate consumers to make purchases. Unlike meat and fish, which tend to have similar colors, placing seasonal and colorful vegetables near the entrance makes the inside of the store look brighter, and can also effectively emphasize the variety and freshness of the store’s products^13^. The effect of such a vegetable section is really reflected in the purchasing behavior of consumers. For example, in a survey of Japanese people aged 20 years and older who purchased vegetables at least once a week, about 90% of participants reported that they used retail stores such as supermarkets and grocery stores^14^. In addition, a previous study that examined key buying factors (KBFs) at the time of vegetable purchase^15^ showed that seasonality ranked third after freshness and price among 12 alternatives. KBFs are defined as the most important issues, reasons, or features that influence a consumer’s decision to purchase a product^16^.

The perception of food-related sensory cues (e.g., flavor and odor) changes with seasons (see review by Spence^7^). An online survey of regular wine consumers in Australia, the United Kingdom, and the United States demonstrated seasonal changes in the preferences for wine-related aromas^17^. Participants preferred wines with a rich chocolate aroma in winter and those with rich aromas of strawberry, passion fruit, lemon, and rose in summer. In a study of German participants, Seo et al.^18^ examined the relationship between odor and time of year (summertime and Christmas season). Their results indicated that the odor of rose was more associated with summertime, while odors of orange, clove, and especially cinnamon were more associated with Christmas season. Some odors differed significantly in terms of familiarity and pleasantness between summertime and Christmas season; for example, cinnamon odor was perceived to be more familiar and pleasant in Christmas season. In addition, the odor of seasonal foods is more commonly experienced at certain times of the year^7,19^. Wada et al.^20^ performed an interesting study on the effects of olfactory cues associated with seasonal foods on emotional responses. More specifically, the ability of infants to associate olfaction with vision was examined using a preferential looking method when strawberries were in or out of season. Two photos (strawberries and tomatoes) were presented side by side to participants who were smelling strawberry odor, and the time spent looking at each photo was measured. Their results indicated that the strawberry photo was preferred when strawberries were in season than when they were out of season. Based on these results^17,18,20^, seasonal foods may provoke some kind of emotional response in consumers.

### Food-evoked nostalgia

Foods have the ability to drive multiple sensations, and may be particularly effective at psychologically transporting consumers back in time^21^. In other words, food can be a valuable mediator of nostalgia^22^. For example, Kim Severson, a correspondent covering food culture in the United States, wrote in the New York Times: “Breakfast cereal is a powerful engine of nostalgia” ^23^. Nostalgia is defined as a positive and balanced complex feeling, emotion, or mood produced by reflection on things (objects, persons, experiences, and ideas) related to the past^24^ and an individual tendency to seek emotional comfort from a familiar past^25^.

Food is an essential part of culture and a common component of community and self-identity^26^. Nostalgic longing and consumption for particular foods sustain culture, familiarity, and self-identity^27^. Furuie^28^ administered a questionnaire survey on local foods for residents of Kitasaku-gun, Nagano, Japan. When participants were asked about the images of local foods, more than 40% of participants responded that those foods were nostalgic. The top ten local foods were six types of dishes containing vegetables, three types of dishes made by boiling fish (carp and crucian carp) or insects (locusts) with seasonings such as soy sauce and sugar, and one type of noodles (*soba*) made from grains. In contrast to meats (beef, pork, chicken, lamb, and mutton), which are essentially unchanged throughout the year, vegetables and fish have seasonality^29^. In addition, there are several popular sayings related to seasonal food in Japan: for example, “eating the catch (or harvest) of the year will extend your life by 75 days” and “summer vegetables cool you down, while winter vegetables warm you up.” Although there is no scientific basis for these popular sayings, Japanese people have long found value in eating seasonal foods. Thus, based on the fact that Japanese food culture places great emphasis on seasonality^30^, there may be some relationship between nostalgic foods and seasonality.

### Nostalgia and autobiographical memories evoked by food odors

While all of our senses may evoke nostalgia, our sense of smell does so most effectively^31^. For example, famous chefs and bartenders devise various ways to evoke nostalgia in their customers through the odor of their dishes and cocktails^32^. A psychological study reported that odors evoked nostalgia to varying degrees in about 54% of trials^33^. On the other hand, music evoked nostalgia in about 26% of trials^34^. In addition, Reid et al.^33^ asked participants not only to evaluate nostalgia for each odor, but also to evaluate the association between the odor and autobiographical memory, and whether the odor resulted in positive or negative emotions. Consequently, odors were classified into three categories: those that evoked nostalgia, those that did not evoke nostalgia but did evoke autobiographical memories, and those that evoked neither nostalgia nor autobiographical memories. The results indicated that odors that evoked nostalgia generated significantly more positive emotions than odors in the other two categories.

The belief that odors are especially evocative of past experiences can be traced back to the literary anecdote “Swann’s Road” by Marcel Proust^35^, in which he vividly recalled his childhood memories when he ate madeleine biscuits dipped in linden tea^36,37^. This experience, referred to as the “Proust phenomenon,” is the basis for the hypothesis that odor-evoked memories are more emotional than those evoked by other sensory stimuli^38^. For example, odors that evoked autobiographical memories were perceived to be significantly more nostalgic than control odors^39^. This finding, as well as those of many previous studies on autobiographical memories evoked by odors^36,40–44^, was obtained in participants who smelled or sniffed the presented odors. However, olfaction is the only dual-sensory modality in the sense that it perceives both objects in the external world and objects in the body (i.e., oral cavity)^45^. Mammals, including humans, have two routes by which odorants reach olfactory receptor neurons: specifically, the orthonasal route, where volatile substances enter the nasal cavity during inhalation and sniffing, and the retronasal route, where food volatiles released in the mouth enter the nasal cavity during exhalation and eating^46^. Both olfactory routes play an important role in modulating food-related behavior and eating experiences^47^. Therefore, in order to maximize control over perceptual attributes that evoke nostalgia and autobiographical memories, we focused on vegetables whose odors participants had experienced both orthonasally and retronasally, that is, those that they had eaten.

### Study goal

Mills and Coleman^48^ stated that nostalgia is a type of autobiographical memory. Autobiographical memory is defined as memory involving the events of one’s life^49^. If autobiographical memories accumulate by experience in daily life, older adults may have more autobiographical memories than young and middle-aged adults^50^. Madoglou et al.^51^ investigated the effect of aging on the representation of nostalgic autobiographical memories in three different age groups (young adults, middle-aged adults, and older adults). Participants answered several questions about emotions generated by recalling past experiences that evoked nostalgia. The older the participants were, the more frequently they experienced evoked nostalgia and the more willing they were to communicate their nostalgic past experiences to others. Therefore, we considered that older adults who had accumulated more autobiographical memories than young and middle-aged adults would be suitable participants in whom to examine the relationship between nostalgic vegetables and seasonality. In addition, a previous study^15^ found that some consumers emphasized seasonality at the time of vegetable purchase (seasonality-oriented; hereafter, SO), while others do not (non-seasonality-oriented; hereafter, non-SO). Accordingly, the relationship between nostalgic vegetables and seasonality may differ between SO and non-SO consumers.

In this study, we hypothesized that SO participants were more likely to feel vegetable-evoked nostalgia than non-SO participants. To test this hypothesis, we performed an online survey on nostalgic vegetables for Japanese older adults. Participants selected KBFs at the time of vegetable purchase from multiple alternatives, including seasonality. After they selected vegetables that they had eaten from among 103 types of vegetables, they further selected nostalgic vegetables from those that they had eaten. In addition, for each nostalgic vegetable, participants evaluated the degree of nostalgia and responded to whether a past event (autobiographical memory) was recalled. If an autobiographical memory was recalled for a certain nostalgic vegetable, psychological characteristics (nostalgia, positive affect, and vividness) of that memory were evaluated. We compared the responses obtained from participants between SO and non-SO participants.

## Methods

### Participants

Through a marketing research company, we recruited potential participants aged 66 years and older who fulfilled the following conditions: (1) participants from all over Japan to eliminate any bias related to birth-place or residence; (2) participants in a gender ratio as close to 50:50 as possible; and (3) having a personal computer at home. The marketing research company sent an email to registered volunteers asking for assistance in an online survey about preference for vegetables. Volunteers who were willing to participate logged into the designated website using an identification (ID) number, which was issued by the marketing research company. The survey ended when 2 weeks had passed after responses began to be accepted. Responses were received from 258 participants aged 66–85 years (110 women and 148 men, average age ± standard deviation [SD] = 71.39 ± 4.75 years).

This study was conducted in accordance with the revised version of the Declaration of Helsinki. All procedures in this study were approved by the ethical committee for ergonomic experiments of the National Institute of Advanced Industrial Science and Technology, Japan (reference number: human 2018-0890-A). We presented an explanation of the survey contents to potential participants at the time of recruitment and just before they began the online survey, and informed them of their right to cease participation even after their initial agreement to participate; all participants provided informed consent by selecting a check button displayed on the computer screen. Procedures for recruitment of participants and ethics conduct in human research were similar to those of a previous study^52^.

### Materials

In this study, we used 103 vegetable names selected for an online survey on preferences for vegetables^52^. The specific selection procedure was as follows. First, we defined vegetables as foods that are sold in the vegetable section of supermarkets and vegetable markets in Japan and that Japanese consumers would be expected to be more likely to recognize as acceptable for inclusion in the vegetable category. Subsequently, five experimenters (four researchers of vegetable-related food manufacturers and one researcher of gustatory and olfactory perception in humans) selected the vegetable names used in this study. More specifically, we selected vegetable names based on two criteria: (1) vegetables that consumers frequently see in stores, such as supermarkets and vegetable markets, and (2) vegetables whose names are likely to be identified by consumers. For the items listed in the vegetable category of *Standards Tables of Food Composition in Japan 2015 (Seventh Revised Edition)*^53^, out of 137 items regarded to be different from the standpoints of use and perceptual attributes, we excluded 50 items with low intake frequency and low familiarity, and adopted the remaining 87 items. Incidentally, tomato, which has long been a topic of debate around the world as to whether it is a vegetable or a fruit^54^, was included in the vegetable category in *Standards Tables of Food Composition in Japan*. Avocado, on the other hand, was one item in the fruit category, but was adopted because it is used like a vegetable. In addition, ten items (*enoki* mushroom, king oyster mushroom, wood ear mushroom, *shiitake* mushroom, *shimeji* mushroom, *nameko* mushroom, oyster mushroom, *maitake* mushroom, white mushroom, and *matsutake* mushroom) were adopted from the mushrooms category. Four items (white potato, sweet potato, taro potato, and Japanese yam) were adopted from the potatoes and starches category. Furthermore, we adopted petite vert, which is not listed in *Standards Tables of Food Composition in Japan*. The 103 vegetable names used in this study are shown in Table S1, categorized by edible part (fruit, leaf I, leaf II, stem, flower, root, and others). Because the category to which leaf vegetables belonged was large, leaf vegetables were arbitrarily divided into two categories (leaf I and leaf II) so that all vegetable names displayed on the screen could be viewed without scrolling during the online survey.

### Procedure

Participants performed the online survey at their own pace at a convenient time, using their home computer. We displayed all instructions related to the online survey on the screen. Participants were instructed to complete their responses in one session, except for cases in which informed consent was withdrawn, as the response could not be saved in the middle of survey. However, participants were allowed to take a short break, such as to use the restroom, as long as the Internet connection was maintained. The time required from start to end of the online survey was up to 30 min. When the participant answered all questions, their responses were sent to a server for data storage.

**Figure 1:**
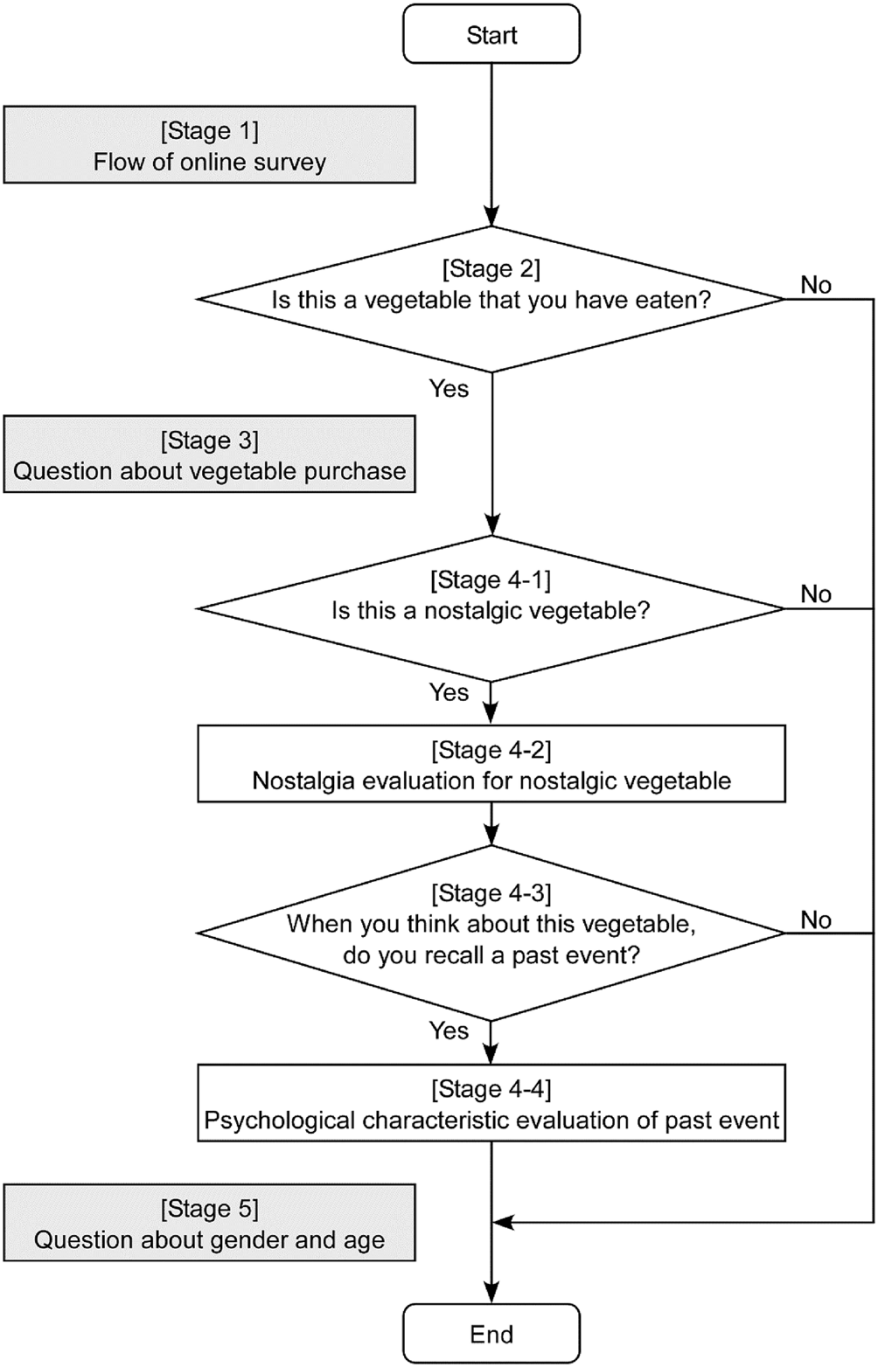
Flowchart for judging and evaluating each vegetable. The diagram illustrates the flow of stages 2, 4-1, 4-2, 4-3, and 4-4 involved in judging and evaluating each vegetable. Stages 1, 3, and 5 did not ask about each vegetable.

Participants accessed the URL of online survey and logged into the website using an ID number sent in advance from the marketing research company. An outline of the online survey is shown in Table 1 and Fig. 1. In stage 1, we explained the flow of online survey. Participants proceeded to stage 2 by clicking the “start” button.

**Table 1:** Outline of online survey on nostalgic vegetables.

Stage	Caption	Content
1	Flow of online survey	“We ask each question in the online survey in the following order. Please answer in order.” Question 1: Selection of vegetables that the participants have eaten Question 2: Question about vegetable purchase Question 3: Question about nostalgic vegetables
2	Selection of vegetables that participants have eaten	“Please select vegetables that you have eaten.” Display of vegetable names Selection of applicable vegetable names
3	Question about vegetable purchase	“Do you buy vegetables yourself? What are the KBFs at the time of vegetable purchase?” Display of “I do not buy vegetables myself” and 10 buying factors Selection of applicable buying factors (multiple responses allowed)
4-1	Question about nostalgic vegetables: nostalgic vegetable selection	“Please select vegetables that evoke nostalgia.” Display of vegetable names that the participants have eaten Selection of applicable vegetable names
4-2	Question about nostalgic vegetables: nostalgia evaluation	“We now ask you about each vegetable that evokes nostalgia. How much nostalgia do you feel from [vegetable name]?” Display of visual analog scale (VAS)
4-3	Question about nostalgic vegetables: recall of past event	“When you think about [vegetable name], do you recall past events?” Selection of response (“yes” or “no”)
4-4	Question about nostalgic vegetables: psychological characteristic evaluation of past event	[Nostalgia]: “How much nostalgia do you feel from the event?” [Positive affect]: “How positive is the impression of the event?” [Vividness]: “How vivid is the memory of the event?” Display of each VAS
5	Question about gender and age	“Please enter your gender and age.” Selection of applicable gender Selection of applicable age

**Figure 2:**
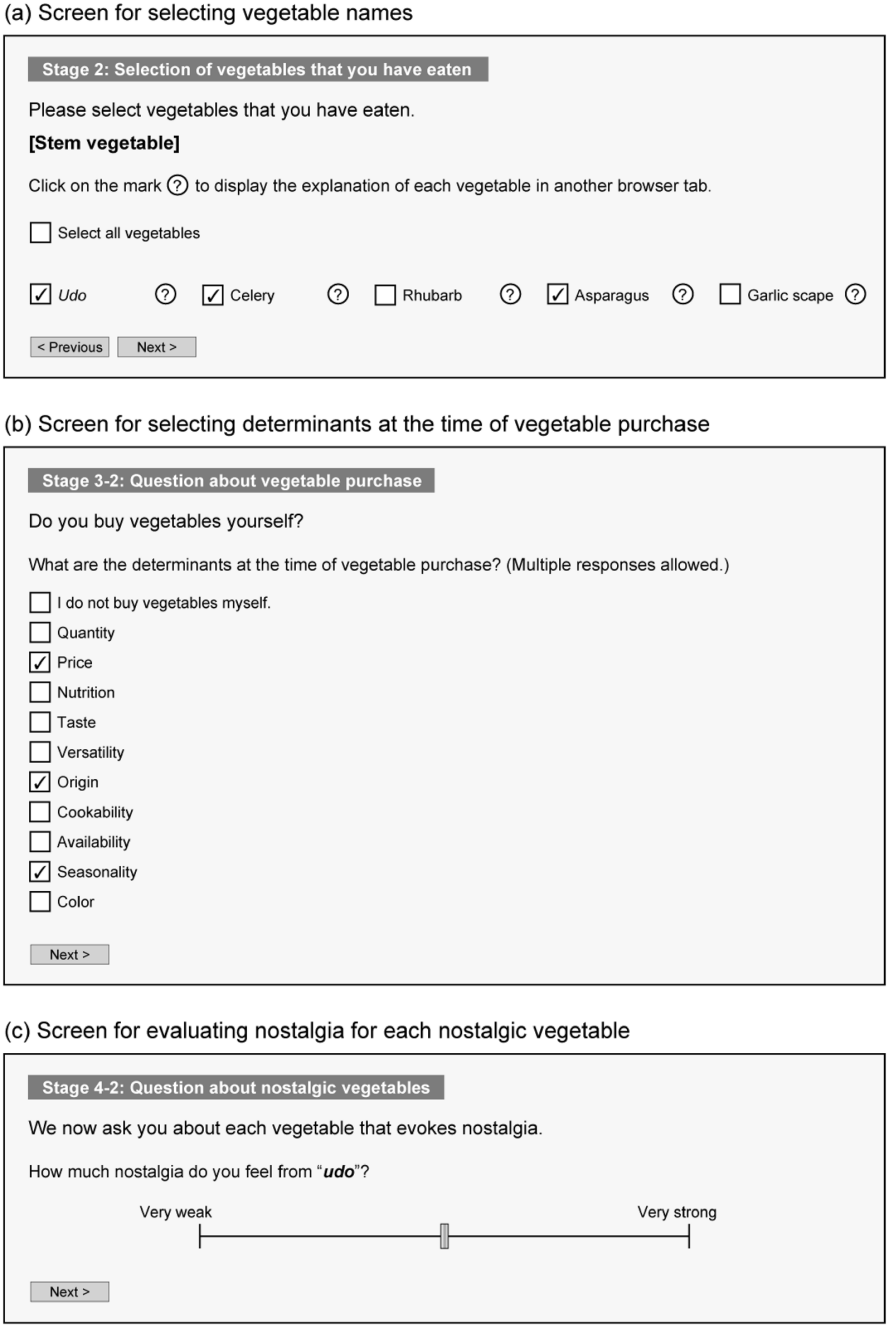
Screen of online survey on nostalgic vegetables. Screens for selecting vegetable names (**a**), for selecting KBFs at the time of vegetable purchase (**b**), and for evaluating various psychological characteristics of nostalgic vegetables (**c**). In (**a**) and (**b**), the participant answered questions by selecting the applicable checkboxes. In addition, in (**a**), when the participant clicked the boxed question mark placed after the vegetable name, another browser tab displayed a brief explanation of that vegetable and its picture. In (**c**), evaluation value was expressed by moving the slider along a visual analog scale.

In stage 2, participants selected vegetables that they had eaten. As shown in Fig. 2a, vegetable names were displayed by switching the screen for each category based on the edible part of the plant. When a participant clicked the boxed question mark placed after the vegetable name, another browser tab displayed a brief explanation of that vegetable and its picture. We randomized the presentation order of vegetable categories and vegetable names within each category among participants. Participants selected applicable checkboxes corresponding to vegetable names. On the screen for selecting vegetable names, the “previous” button (except on the screen for the first category) and the “next” button were displayed. On the final screen of stage 2, we displayed alternatives other than those selected as vegetables that the participant had eaten, the “previous” button, and the “next” button. We suspected that for most participants, the number of vegetables that they had never eaten was smaller than the number of those that they had. Hence, in order to easily confirm the selected alternatives, we decided to display the vegetable names that were not selected as vegetables that they had eaten. If participant selected all vegetables, we displayed the message “all vegetables were selected as vegetables that you have eaten.” If the participants clicked the “next” button on this screen, they could not change the responses of stage 2 and proceeded to stage 3-1. If there were no vegetables that the participant had eaten, the participant proceeded to stage 5 by clicking the “next” button. The procedure up to stage 2 was the same as in a previous study^52^.

In stage 3, as shown in Fig. 2b, we asked whether participants had bought vegetables themselves, and if so, the KBFs at the time of vegetable purchase. More specifically, participants who bought vegetables themselves selected applicable checkboxes corresponding 10 buying factors (quantity, price, nutrition, taste, versatility, origin, cookability, availability, seasonality, and color). At least one alternative had to be selected. Participants proceeded to stage 4-1 by clicking the “next” button.

In stage 4-1, participants selected nostalgic vegetables. For each participant, only the names of vegetables selected as eaten in stage 2 were used, and vegetable names were displayed by switching the screen for the category based on the edible part. We randomized the presentation order of vegetable categories and vegetable names within each category. Participants selected the applicable checkboxes corresponding to vegetable names. On the final screen of stage 4-1, we displayed all alternatives selected as nostalgic vegetables, the “previous” button, and the “next” button. If participants clicked the “next” button on this screen, they could not change the responses of stage 4-1 and proceeded to stage 4-2. If there were no nostalgic vegetables, we displayed the message “you had no nostalgic vegetables,” and the participant proceeded to stage 5 by clicking the “next” button.

In stage 4-2, vegetable names selected in stage 4-1 were displayed in random order. Referring to a study by Yamamoto^55^, participants were asked to evaluate the degree of nostalgia for each nostalgic vegetable. As shown in Fig. 2c, they used a visual analog scale (VAS) with verbal labels of “very weak” and “very strong” on the left and right ends, respectively. Participants indicated the evaluation value by moving the slider, which was displayed in the center of the scale as the initial value, along the scale. Participants proceeded to stage 4-3 by clicking the “next” button.

In stage 4-3, after participants answered whether an autobiographical memory was recalled for the nostalgic vegetable name displayed in stage 4-2 by selecting the “yes” or “no” radio button, they clicked the “next” button. If they selected the “yes” radio button, they proceeded to stage 4-4. If they selected the “no” radio button, they returned to stage 4-2, and the degree of nostalgia was evaluated for the next nostalgic vegetable name.

In stage 4-4, referring to a study by Yamamoto^55^, participants were asked to evaluate the degree of nostalgia for the autobiographical memory related to a nostalgic vegetable, and were also asked to evaluate the degree of positive affect and vividness for that memory. The same scale as in stage 4-2 was used to evaluate each psychological characteristic of the autobiographical memory. If all vegetable names selected in stage 4-1 were presented, participants proceeded to stage 5; otherwise, they returned to stage 4-2.

In stage 5, participants entered their gender (female or male) and age. They selected gender with a radio button and age from a drop-down list. When the participant clicked the “end” button, we displayed the message “we appreciate your cooperation”; at the same time, all responses obtained from the participant were sent to the server.

### Analysis

#### Selection of data used for analysis

We excluded 63 participants who responded that they did not buy vegetables themselves from the analyses. Responses obtained from 195 participants (109 women, 86 men, average age ± SD = 71.06 ± 4.61 years) were included in the analyses.

#### Proportion of participants with or without emphasis on each buying factor

For each buying factor (quantity, price, nutrition, taste, versatility, origin, cookability, availability, seasonality, and color) at the time of vegetable purchase, participants were categorized as KBF or non-KBF groups by whether they did or did not emphasize a given factor. The number of participants in each group for each buying factor is shown in Table 2.

**Table 2:** Number of participants in the KBF and non-KBF groups for each buying factor at the time of vegetable purchase.

Buying factor	KBF group	Non-KBF group	*p* value
Quantity	94 (0.42)	101 (− 0.42)	0.672
Price	158 (10.11)	37 (− 10.11)	< 0.001***
Nutrition	92 (0.12)	103 (− 0.12)	0.904
Taste	73 (− 2.75)	122 (2.75)	0.006**
Versatility	39 (− 7.90)	156 (7.90)	< 0.001***
Origin	106 (2.24)	89 (− 2.24)	0.025*
Cookability	71 (− 3.06)	124 (3.06)	0.002**
Availability	53 (− 5.78)	142 (5.78)	< 0.001***
Seasonality	143 (7.84)	52 (− 7.84)	< 0.001***
Color	83 (− 1.24)	112 (1.24)	0.215

Numerical values in parentheses are adjusted standardized residuals, and *p* values (two-sided) are results of residual analyses. ***p < 0.001, **p < 0.01, *p < 0.05.

To determine whether there was a difference among the buying factors in the proportion of KBG group to non-KBF group, we performed the chi-square test for a two-way table (10 buying factors × 2 groups). Based on the significance of this result, an analysis of adjusted standardized residuals (two-tailed test) was performed to identify buying factors emphasized by significantly more or fewer participants in the KBF group.

#### Proportion of participants with or without nostalgic vegetables

For each buying factor and each group (KBF and non-KBF), we calculated the number of participants who identified at least one nostalgic vegetable and the number of those who identified none. These results are stratified by buying factor in Table 3. To determine whether there was a difference between the KBF and non-KBF groups in the proportion of participants who identified nostalgic vegetables to those who did not, we performed the chi-square test for each buying factor.

**Table 3:** Number of participants with and without nostalgic vegetables in the KBF and non-KBF groups for each buying factor at the time of vegetable purchase.

Buying factor	KBF group		Non-KBF group		*χ*^*2*^ value	*p* value
	With NV	Without NV	With NV	Without NV		
Quantity	75	19	75	26	0.84	0.360
Price	124	34	26	11	1.14	0.286
Nutrition	74	18	76	27	1.21	0.271
Taste	58	15	92	30	0.42	0.517
Versatility	31	8	119	37	0.18	0.671
Origin	85	21	65	24	1.40	0.238
Cookability	58	13	92	32	1.43	0.232
Availability	43	10	107	35	0.73	0.394
Seasonality	118	25	32	20	9.45	0.002**
Color	69	14	81	31	3.14	0.076

NV: nostalgic vegetable. *df* (degree of freedom) = 1. **p < 0.01.

#### Numbers of nostalgic vegetables with or without associated autobiographical memories

To examine the effect of orientation toward seasonality on the number of nostalgic vegetables with and without associated autobiographical memories, we performed two-way mixed-design analysis of variance (ANOVA) with seasonality subgroup (SO and non-SO; that is, corresponding to participants in the KBF and non-KBF groups regarding seasonality) as the between-subject factor and autobiographical memory (with and without) as the within-subject factor, using the number of nostalgic vegetables reported by each participant. A simple effects test was performed based on the significance of results obtained with ANOVA.

#### Nostalgia for nostalgic vegetables with or without associated autobiographical memories

In the SO subgroup, 97 of 143 participants reported nostalgic vegetables with associated autobiographical memories; 1–17 types of vegetables were reported per participant, resulting in a total of 351 types, including duplicates. In addition, 60 participants reported nostalgic vegetables without associated autobiographical memories; 1–10 types of vegetables were reported per participant, resulting in a total of 185 types, including duplicates. Thirty-nine participants reported both nostalgic vegetables with and without associated autobiographical memories.

In the non-SO subgroup, 24 of 52 participants reported nostalgic vegetables with associated autobiographical memories; 1–11 types of vegetables were reported per participant, resulting in a total of 76 types, including duplicates. In addition, 18 participants reported nostalgic vegetables without associated autobiographical memories; 1–9 types of vegetables were reported per participant, resulting in a total of 74 types, including duplicates. Ten participants reported both nostalgic vegetables with and without associated autobiographical memories.

For all nostalgic vegetables, with or without associated autobiographical memories, we regard the left and right ends of the VAS regarded as 0 and 1 and calculated the evaluation value of nostalgia. To examine the effects of orientation toward seasonality and autobiographical memories on nostalgia for nostalgic vegetables, we performed two-way factorial ANOVA with seasonality subgroup (SO and non-SO) and autobiographical memory (with and without) as the between-subjects factors. A simple effects test was performed based on the significance of results obtained with ANOVA.

#### Psychological characteristics of autobiographical memories

For the 351 and 76 nostalgic vegetables with associated autobiographical memories reported by participants in SO and non-SO subgroups, respectively, we regarded the left and right ends of the VAS as 0 and 1 and calculated the evaluation values of nostalgia, positive affect, and vividness. To examine the effect of orientation toward seasonality on psychological characteristics of nostalgic vegetable-related autobiographical memories, unpaired *t*-tests were performed for each psychological characteristic.

We used *Ekuseru-Toukei* 2012 (Social Survey Research Information, Tokyo, Japan) for statistical analysis, and the significance level was set at 5%.

## Results

### Proportion of participants with or without emphasis on each buying factor

The chi-square test revealed a significant difference among the buying factors in the proportion of KBF group to non-KBF group (*χ*^2^ (9) = 254.68, *p* < 0.001). Residual analysis revealed that the KBF group had significantly more participants for price, origin, and seasonality, but significantly fewer participants for taste, versatility, cookability, and availability (see Table 2 for details).

### Proportion of participants with or without nostalgic vegetables

The chi-square test for each buying factor revealed that only for seasonality, the KBF and non-KBF groups differed significantly regarding the proportion of participants who identified nostalgic vegetables to those who did not; that is, there was a significant difference between the SO and non-SO subgroups (see Table 3 for details). This result demonstrated that the SO subgroup identified significantly more nostalgic vegetables than the non-SO subgroup.

### Numbers of nostalgic vegetables with or without associated autobiographical memories

**Figure 3:**
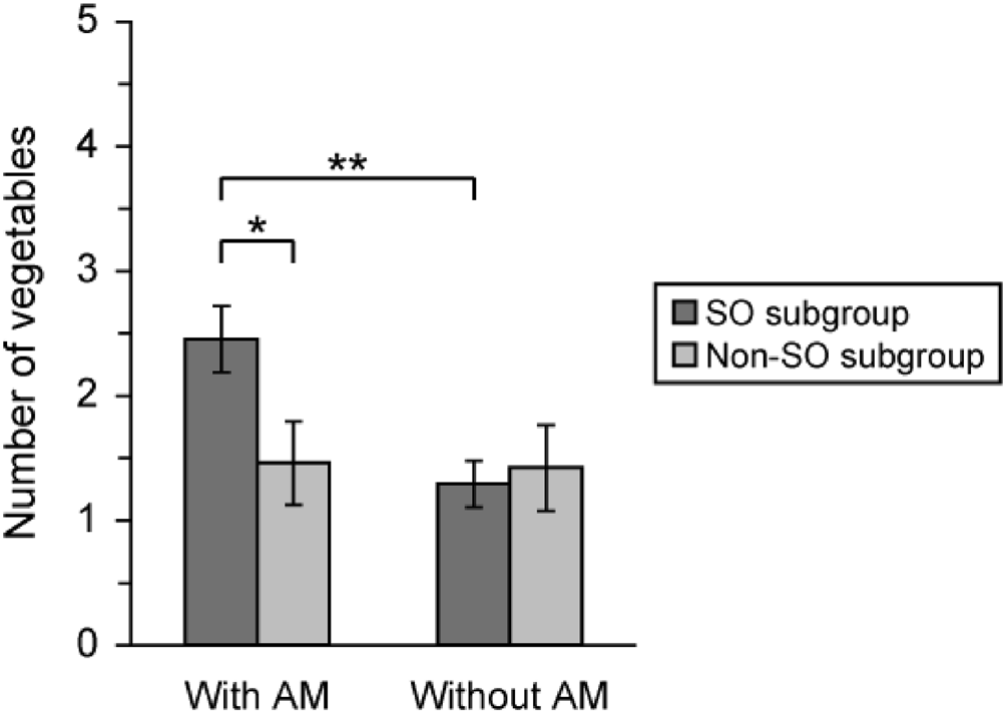
Number of nostalgic vegetables with and without autobiographical memories in each seasonality subgroup. Number of nostalgic vegetables with and without associated autobiographical memories in the SO and non-SO subgroups. Two-way mixed-design ANOVA revealed a significant interaction between seasonality subgroup and autobiographical memory (*F* (1, 193) = 5.10, *p* < 0.05). AM: autobiographical memory. Error bar: standard error (SE). ***p < 0.001, *p < 0.05.

The number of nostalgic vegetables with and without associated autobiographical memories in each seasonality subgroup is shown in Fig. 3. Two-way mixed-design ANOVA revealed a significant main effect of autobiographical memory (*F* (1, 193) = 5.83, *p* < 0.05) and a significant interaction between seasonality subgroup and autobiographical memory (*F* (1, 193) = 5.10, *p* < 0.05). The simple effects test for interaction revealed a significant simple main effect of seasonality subgroup for nostalgic vegetables with associated autobiographical memories (*F* (1, 386) = 5.30, *p* < 0.05), and a significant simple main effect of autobiographical memory in the SO subgroup (*F* (1, 193) = 10.92, *p* < 0.01).

These results demonstrated that the number of nostalgic vegetables with associated autobiographical memories was significantly higher in the SO subgroup than in the non-SO subgroup, and that the SO subgroup had a greater number of nostalgic vegetables with associated autobiographical memories than those without associated autobiographical memories.

### Nostalgia for nostalgic vegetables with or without autobiographical memories

**Figure 4:**
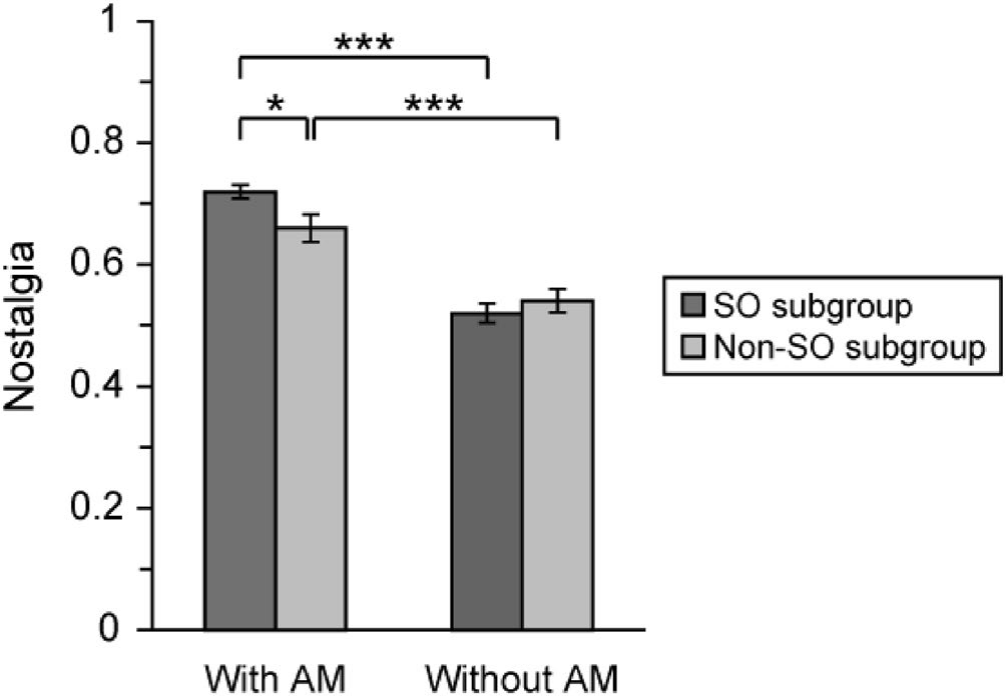
Nostalgia for nostalgic vegetable with and without autobiographical memories in each seasonality subgroup. Nostalgia for nostalgic vegetables with and without associated autobiographical memories in the SO and non-SO subgroups. Two-way factorial ANOVA revealed a significant interaction between seasonality subgroup and autobiographical memory (*F* (1, 682) = 4.38, *p* < 0.05). AM: autobiographical memory. Error bar: SE. ***p < 0.001, **p < 0.01, *p < 0.05.

Nostalgia for nostalgic vegetables with and without associated autobiographical memories in each seasonality subgroup is shown in Fig. 4. Two-way factorial ANOVA revealed a significant main effect of autobiographical memory (*F* (1, 682) = 69.52, *p* < 0.001) and a significant interaction between seasonality subgroup and autobiographical memory (*F* (1, 682) = 4.38, *p* < 0.05). The simple effects test for interaction revealed a significant simple main effect of seasonality subgroup for nostalgic vegetables with associated autobiographical memories (*F* (1, 682) = 5.26, *p* < 0.05), and significant simple main effects of autobiographical memory in the SO subgroup (*F* (1, 682) = 115.10, *p* < 0.001) and in the non-SO subgroup (*F* (1, 682) = 12.77, *p* < 0.001).

These results indicated that nostalgia was significantly higher for nostalgic vegetables with associated autobiographical memories in the SO subgroup than for nostalgic vegetables without associated autobiographical memories in the SO subgroup and for nostalgic vegetables with associated autobiographical memories in the non-SO subgroup. In addition, nostalgia in the non-SO subgroup was significantly higher for nostalgic vegetables with associated autobiographical memories than for those without associated autobiographical memories.

### Psychological characteristics of autobiographical memories

**Figure 5:**
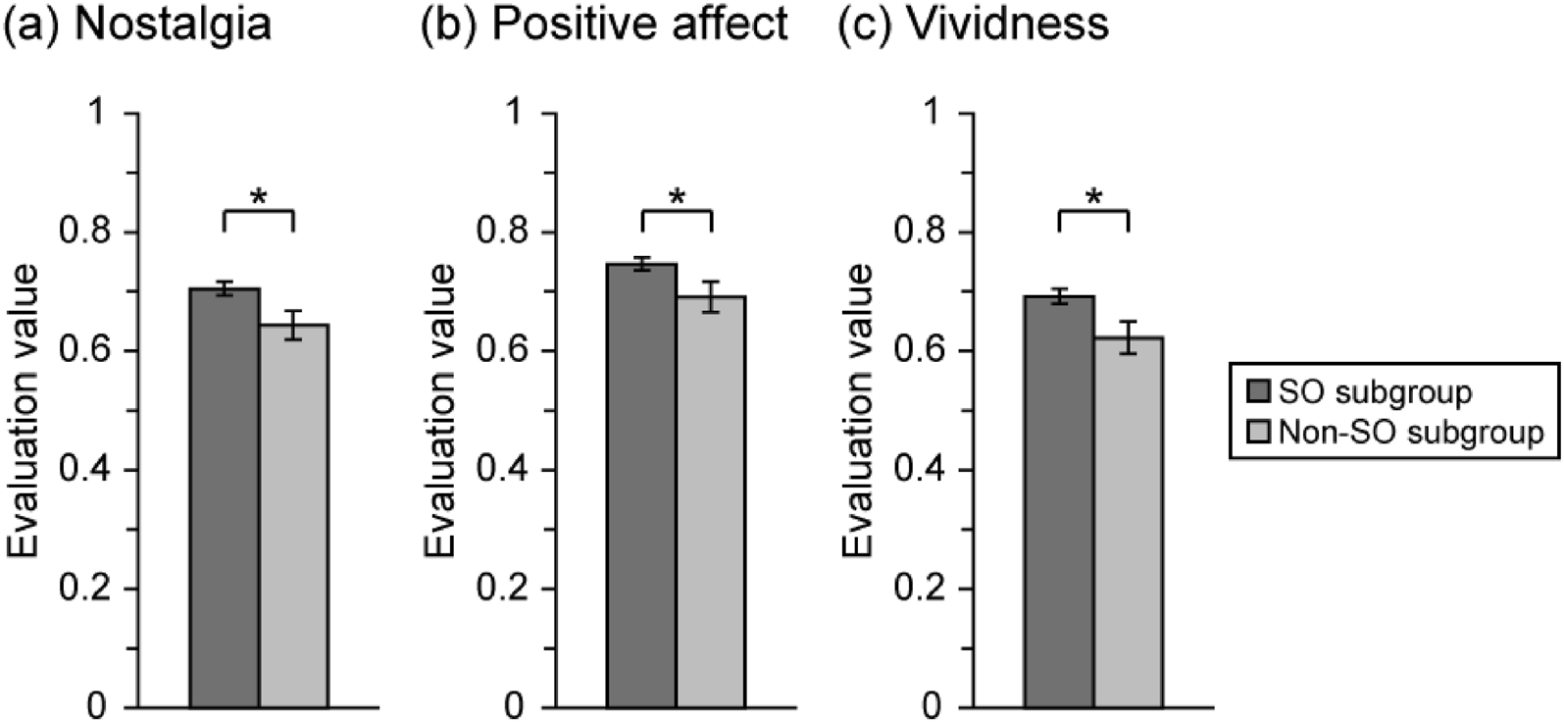
Psychological characteristics of nostalgic vegetable-related autobiographical memories in each seasonality subgroup. Nostalgia (**a**), positive affect (**b**), and vividness (**c**) for nostalgic vegetable-related autobiographical memories in the SO and non-SO subgroups. Unpaired *t*-test revealed significant differences between the two groups in nostalgia (*t* (425) = 2.27, *p* < 0.05), positive affect (*t* (425) = 2.14, *p* < 0.05), and vividness (*t* (425) = 2.28, *p* < 0.05). Error bar: SE. *p < 0.05.

Nostalgia, positive affect, and vividness for nostalgic vegetable-related autobiographical memories in the SO and non-SO subgroups are shown in Fig. 5. Unpaired *t*-tests for each psychological characteristic revealed significant differences between the two groups in nostalgia (*t* (425) = 2.27, *p* < 0.05), positive affect (*t* (425) = 2.14, *p* < 0.05), and vividness (*t* (425) = 2.28, *p* < 0.05). These results indicated that the SO subgroup had significantly higher nostalgia, positive affect, and vividness than the non-SO subgroup.

## Discussion

### KBFs at the time of vegetable purchase

Consumers’ purchase intentions are influenced by various factors [see review by Pandey and Srivastava^56^ for details]. Purchase intention can be investigated from the perspective of food properties in which consumers have interests at the time of purchase^57^. In this study, 10 alternatives (quantity, price, nutrition, taste, versatility, origin, cookability, availability, seasonality, and color) were evaluated as potential KBFs at the time of vegetable purchase. Of these, price, origin, and seasonality were significantly more likely than the others to be emphasized by participants at the time of vegetable purchase.

Price-oriented and origin-oriented purchase behavior was also reported in a study by Brumfield et al.^58^. They performed a face-to-face interview during tomato season at a supermarket in New Jersey to identify factors that affected tomato purchases. Their results demonstrated that higher price was correlated with lower rates of purchase. However, the effect of price was significantly smaller for tomatoes from New Jersey than for tomatoes from other regions. In addition, participants increased their purchase amounts if the tomatoes were from New Jersey, but decreased purchase amounts if the tomatoes were from other regions. This origin-oriented purchase behavior was also observed in a study of Japanese consumers by Murata et al.^59^. They asked participants whether they would select a high-priced domestic food or low-priced foreign food displayed in the fresh food section. Their results demonstrated that 85% of all participants were domestic-oriented, and that the older the group, the higher the proportion of domestic-oriented participants. In another consumption trend survey on vegetables^15,60^, the proportion of price-oriented participants was high regardless of age, and the proportions of origin-oriented and seasonality-oriented participants increased with age. Accordingly, the finding in this study that significantly more participants used price, origin, and seasonality as KBFs at the time of vegetable purchase was consistent with the results of previous studies^15,58–60^.

### Nostalgic vegetables and orientation toward seasonality

In this study, we hypothesized that the SO subgroup was more likely to feel vegetable-evoked nostalgia than the non-SO subgroup. Among the 10 alternatives defined as potential KBFs at the time of vegetable purchase, only seasonality had significantly more participants who identified nostalgic vegetables in the KBF group than in the non-KBF group. In addition, when nostalgic vegetable-related autobiographical memories were recalled, the number of nostalgic vegetables, nostalgia for nostalgic vegetables, and nostalgia for autobiographical memories were significantly higher in the SO subgroup than in the non-SO subgroup. On the other hand, when nostalgic vegetable-related autobiographical memories were not recalled, the number of nostalgic vegetables and the nostalgia for nostalgic vegetables were similar between the SO and non-SO subgroups. These results supported our hypothesis.

The important differences in nostalgic vegetables between the SO and non-SO subgroups were caused by vegetables with associated autobiographical memories. Vegetables are mostly seasonal, and their production is limited by local climate^61,62^. For example, summer vegetables will not be on the market until the following summer when the season is over. Consumers who emphasize seasonality may perceive the passage of time based on the types of vegetables displayed in stores. Hwanga and Hyun^63^ identified four triggers that evoked nostalgia in patrons in luxury restaurants: (1) foods served during the restaurant visit, (2) memorable events experienced in the restaurant, (3) unique restaurant environment (interior or exterior design), and (4) staff members with whom they communicated previously in the restaurant. The nostalgia elicited positive emotional responses (i.e., pleasure), and the positive emotional responses stimulated intentions to revisit. The longer the period without a visit, the more effectively nostalgia elicited positive emotional responses. Based on the results of a study by Hwanga and Hyun^63^, we considered that because the SO subgroup always recognized that seasonal vegetables could only be obtained at a limited time of the year, they might express stronger emotional responses than the non-SO subgroup when they saw seasonal vegetables in stores such as supermarkets and vegetable markets. Such an emotional response may have been one of the triggers for recall of autobiographical memory.

With regard to psychological characteristics of nostalgic vegetable-related autobiographical memories, not only nostalgia but also positive affect and vividness were significantly higher in the SO subgroup than in the non-SO subgroup. Previous studies^64–69^ reported that nostalgia had a positive relationship with positive affect. Odors that evoked nostalgia elicited significantly more positive emotions than odors that recalled autobiographical memories but not nostalgia or odors that evoked neither nostalgia nor autobiographical memories^33^. On the other hand, Oba et al.^70^, who examined factors that influence nostalgia using visual stimuli (photographs) related to elementary school days, reported that vividness did not predict intensity of nostalgia. Oba et al. explained this observation as follows: first, general (rather than specific) visual stimuli were used, and second, participants were not explicitly asked to recall their specific autobiographical memories from their elementary school days. In this study, participants were not asked to recall memories of a specific period in their lives, but instead voluntarily selected vegetables that evoked nostalgia. Therefore, the SO subgroup may have been able to recall nostalgic vegetable-related autobiographical memories more nostalgically, positively, and vividly than the non-SO subgroup. We speculate that this also affected nostalgia for nostalgic vegetables and the number of nostalgic vegetables with associated autobiographical memories.

### Expected role of vegetable-evoked nostalgia

The aging of the Japanese population is unprecedented in the world^71^. Living with children was once very common among Japanese older adults, but recently the living environment of people aged 65 and over has changed dramatically^72^. Single-person households consisting of older adults are becoming more common, regardless of gender: in 1980, the proportion of single-person households in the population over the age of 65 was 4.3% among men and 11.2% among women; in 2015, it was 13.3% among men and 21.1% among women; and in 2040, it is expected to be 20.8% among men and 24.5% among women^73^. In addition, according to a fact-finding survey on life-styles and eating habits of older Japanese adults, about 96% of individuals in single-person households sometimes or exclusively ate alone^74^. Solitary eating can have a negative impact on people’s nutritional status and physical health^75–77^, and also their psychological well-being^78,79^.

Humans are thoroughly social creatures^80^. Living alone, social isolation, and loneliness are independent events, but there are causal relationships between these events^81^. Social isolation can be structurally defined as the absence of social interactions, contacts, relationships with family and friends, relationships with neighbors at the individual level, and relationships with “society at large” at a broader level^82^. Zhou et al.^69^ described two different pathways for the effect of loneliness on perceived social support. The first was a direct pathway in which loneliness decreased perceived social support. In other words, if loneliness is reduced, perceived social support is likely to improve. For example, eating with others can effectively reduce loneliness in older adults^83^. In recent years, digital technology has rapidly developed to connect solo diners with physically distant or virtual dining partners^84^. The second was an indirect pathway in which loneliness improved perceived social support via nostalgia. Their findings suggest that nostalgia defused a negative emotion (loneliness) and produced a positive result (promotion of perceived social support).

Locher et al.^27^ considered nostalgic foods to be a category of comfort foods. Comfort food is food that provides consolation and a feeling of well-being through its consumption, and it tends to be positively associated with a specific person, place, or time^85^. Therefore, when a comfort food is served to older adults, they may feel healed or cared for and remember pleasant experiences of their childhood^86^. Troisi and Gabriel^87^ reported that comfort foods defused loneliness of participants with a secure attachment style who have positive cognitive associations with human relationships. In this study, we found that the SO subgroup was more likely than the non-SO subgroup to feel vegetable-evoked nostalgia and to recall nostalgic vegetable-related autobiographical memories. These results suggest that consumers who emphasize seasonality perceive vegetables as comfort foods. If so, vegetables may become comfort foods in the future even for consumers who do not currently emphasize seasonality if they repeatedly use seasonality as a KBF at the time of vegetable purchase (i.e., seasonality-conscious purchasing behavior). However, future research is necessary to determine if nostalgia evoked by seasonal vegetables actually enhances the psychological well-being of older adults.

### Limitations of this study and future issues

Participants selected vegetables that evoked nostalgia from among those that they had eaten. However, even vegetables that one has seen but never eaten can evoke nostalgia and autobiographical memories. If participants select nostalgic vegetables from a list of vegetables and are then asked whether they have eaten each one, we can collect data on both nostalgic vegetables that they have and have not eaten.

Participants identified the KBFs at the time of vegetable purchase, and were categorized based on their responses. It may have been more straightforward to examine nostalgia and autobiographical memories evoked by those that they had purchased, rather than vegetables that they had eaten. In addition, if we were to focus on seasonality in particular among the KBFs at the time of vegetable purchase, it would have been informative to perform a preliminary survey to determine which vegetables evoke seasonality and to what extent. Based on the results, we could have used only the names of vegetables associated strongly with seasonality.

In this study, we focused on nostalgia and autobiographical memories evoked by vegetables as ingredients rather than as a part of dish. However, Spence^32^ stated that while food-related neural activity is most intense when we anticipate a food, it is the long-lasting memory of a special meal that gives us great long-term pleasure. What we encounter at mealtime is commonly the finished dish, not the uncooked ingredients. In a survey of Japanese adults on nostalgia-evoked foods, participants identified 40 items, of which 95% (38 items other than Japanese plums and whale meat) were names of dishes or commercial products^55^. Based on these previous studies^32,55^, it is possible that specific dishes using vegetables, rather than vegetables as an ingredient, trigger more vivid nostalgia and autobiographical memories. For some Japanese people, for example, French fries may simply make them think of the menu of a fast food restaurant, while “*nikujaga*” (meat and potatoes stewed in a sweet soy sauce-based broth) may be nostalgic as a taste of mom’s cooking.

Japanese retailers who deal in vegetables want to avoid seasonal supply interruptions^88^. Currently, the intensification of agriculture, use of new technologies, extension of natural production and growing seasons, and increases in international trade have made it possible to provide a year-round supply of fresh agricultural products^4^. For example, in terms of domestic production, facility cultivation (e.g., using plastic greenhouses) permits easier temperature control than open-field cultivation and contributes to a stable supply of vegetables^89^. In addition, most imported vegetables comprise those used to make up for supply shortages during the off-season, those that cannot be grown in Japan, and those whose domestic production is decreasing^90^. According to Japan’s agriculture, forestry, and fisheries product statistics for 2020, the largest percentage of imported fresh vegetables came from neighboring China, followed by New Zealand^91^. Producers in countries and regions of the southern hemisphere, such as New Zealand, whose seasons are opposite to those of Japan, supply vegetables during Japan’s agricultural off-season^88^. Thus, as seasonal variation in vegetable distribution has decreased, the perceived seasonality of vegetables (i.e., local seasonality) may have also become more ambiguous. However, according to a survey of university students and their parents on the seasonality of vegetables^92^, 93% of parents and 48% of university students took seasonality into account when planning their daily menus. In other words, they found that about half of young participants were concerned with vegetable seasonality, even though in recent years it has become much more common for vegetables to be supplied year-round. Children and adolescents can learn about the seasonality of foods through meals at home, school lunches, and school classes (nutrition education). If they go to the supermarket, they can find point-of-purchase advertisements promoting seasonal foods in the vegetable section. Therefore, as long as such experiences are not lost, the relationship between nostalgic vegetables and seasonality will probably be maintained to some extent, even as time progresses or generations change. This hypothesis requires confirmation in the future.

Finally, the results of this study may be less applicable in countries or regions where seasonality is unlikely to be a KBF at the time of food purchase or where the variety of vegetables on sale is relatively small. However, our methodology can be applied to KBFs other than seasonality (e.g., origin) and to food categories other than vegetables (e.g., fruits).

## Conclusion

SO participants felt more vegetable-evoked nostalgia than non-SO participants. The important differences between SO and non-SO participants were caused by nostalgic vegetables with associated autobiographical memories. Orientation toward seasonality may have elicited nostalgic vegetable-related autobiographical memories and emotional responses to those memories, which in turn increased the number of nostalgic vegetables with associated autobiographical memories and the nostalgia for nostalgic vegetables. The findings of this study suggest that seasonality-conscious purchasing behavior evokes nostalgia, but were obtained from an online survey using vegetable names. For more robust evidence, intervention experiments would be needed to demonstrate that actually purchasing and consuming seasonal vegetables enhances nostalgia.

## Supplementary Information

Supplementary Information 1.

Supplementary Information 2.

Supplementary Table S1.

## Data Availability

The data used to generate the results that support the findings of this study are included in the Supplementary Information.
