# Supplementary material for: Nostalgia evocation through seasonality-conscious purchasing behavior revealed by online survey using vegetable names
Source: Sci Rep. 2022 Apr 2;12:5568. doi: 10.1038/s41598-022-09485-2 (PMC8976854; doi:10.1038/s41598-022-09485-2)
Supplement: Supplementary file 3 — Supplementary Table S1. [file 41598_2022_9485_MOESM3_ESM.docx]

Table S1: Vegetable name for each category based on edible part, used for online survey

| Name | Name | Name |
| --- | --- | --- |
| Fruit vegetables | Parsley | Root vegetables |
| Avocado | Potherb mustard | Bamboo shoot |
| Bell pepper | Radish sprout | Carrot |
| Bitter gourd | Shepherd’s purse | Garlic |
| Cherry tomato | Shiso | Ginger |
| Cucumber |  | Japanese burdock |
| Eggplant | Leaf vegetables II | Japanese radish |
| Fava bean | Cabbage | Japanese shallot |
| Green bean | Chinese chive | Japanese yam |
| Green pea | Garland chrysanthemum | Lotus root |
| Green soybean | Japanese green onion | Onion |
| Okra | Japanese mugwort | Radish |
| Peanut | Japanese mustard spinach | Red beet |
| Podded pea | Japanese parsley | Red onion |
| Red bell pepper | Japanese turnip green | Shallot |
| Squash | Kale | Sweet potato |
| Sweet corn | Leaf green onion | Taro potato |
| Sweet green pepper | Nalta jute | Turnip |
| Tomato | Pea sprout | White potato |
| White gourd-melon | Petit vert |  |
| Yellow bell pepper | Red cabbage | Others |
| Zucchini | Saltwort | Aloe |
|  | Small green onion | Bean sprout |
| Leaf vegetables I | Spinach | Brussels sprout |
| *Angelica keiskei* | Spring onion | Dried gourd shaving |
| Arugula | Watercress | *Enoki* mushroom |
| Basil |  | Japanese butterbur |
| Bok choy | Stem vegetables | Japanese ginger |
| Boston lettuce | Asparagus | King oyster mushroom |
| Chicory | Celery | *Maitake* mushroom |
| Chinese cabbage | Garlic scape | *Matsutake* mushroom |
| Coriander | Rhubarb | *Nameko* mushroom |
| Japanese chive | *Udo* | Oyster mushroom |
| Japanese honewort |  | *Shiitake* mushroom |
| Japanese leaf mustard | Flower vegetables | *Shimeji* mushroom |
| Japanese radish leaves | Artichoke | White mushroom |
| Leaf mustard | Broccoli | Wood ear mushroom |
| Lettuce | Canola flower | Young corn |
|  | Cauliflower |  |
